# Metformin Regulates TET2 Expression to Inhibit Endometrial Carcinoma Proliferation: A New Mechanism

**DOI:** 10.3389/fonc.2022.856707

**Published:** 2022-04-11

**Authors:** Jingbo Zhang, Lei Kuang, Yanyu Li, Qing Wang, Hui Xu, Jianwei Liu, Xueyan Zhou, Yang Li, Bei Zhang

**Affiliations:** ^1^ Department of Obstetrics and Gynecology, Xuzhou Central Hospital, Xuzhou Clinical School of Xuzhou Medical University, Xuzhou, China; ^2^ Jiangsu Key Laboratory of New Drug Research and Clinical Pharmacy, Xuzhou Medical University, Xuzhou, China; ^3^ Xuzhou Institute of Medical Science, Xuzhou, China

**Keywords:** TET2, 5hmC, metformin, endometrial cancer, AMPK

## Abstract

**Objectives:**

To investigate the relationship between TET2 expression and endometrial cancer’s clinicopathological features and prognosis, and the effect of metformin on TET2 and 5hmC levels in endometrial cancer cells.

**Methods:**

The clinical significance of TET2 expression in endometrial carcinoma was analyzed from TCGA public database. Eighty-eight patients with endometrial cancer and 20 patients with normal proliferative endometrium were enrolled in this study. TET2 and 5hmC were respectively detected by Immunohistochemistry and ELISA in endometrial tissues. Kaplan-Meier and Cox proportional hazard regression models were used to analyze relationships between TET2 and 5hmC and the overall survival of EC patients. Endometrial cell proliferation was assessed after TET2 gene knockdown. Western blotting and real-time PCR were used to detect the effect of metformin on TET2 expression and to explore whether AMPK is involved in metformin-mediated TET2 regulation.

**Results:**

The clinical significance of expression of TET2 in endometrial cancer from TCGA public database confirmed that TET2 expression was significantly down-regulated in cancer samples and TET2 expression was also significantly different among different histopathological samples and TET2 is down-regulated in advanced, high-grade, and relapsed endometrial carcinoma tissues(P<0.05). Immunohistochemical analysis showed that TET2 and 5hmC levels were significantly lower in endometrial adenocarcinoma(P<0.05). TET2 expression was correlated with the degree of EC differentiation (P < 0.05). 5hmC levels were associated with clinical stage, differentiation, the depth of myometrial invasion, and lymph node metastasis (P < 0.05). The mean survival time of patients with negative staining for TET2 and 5hmC was shorter than that of patients with positive staining for both markers (P<0.05). Multivariate Cox regression analysis showed that TET2 expression was an independent risk factor for prognosis in patients with endometrial adenocarcinoma (HR = 14.520, 95% CI was 1.From 060 to 198.843, P = 0.045). siRNA-mediated TET2 knockdown increased the proliferation of EC cells. Metformin increased the levels of TET2 and 5hmC in EC cells. AMPK was involved in the regulation of TET2 by metformin.

**Conclusions:**

TET2 may play an important role in EC development and may be a prognostic marker. Moreover, TET2 may be involved in a novel mechanism by which metformin inhibits EC cell proliferation.

## Introduction

Endometrial cancer (EC) is one of the most common gynecological malignancies worldwide (1). The development of EC is a multistep process involving many molecular biological changes. EC has been shown to be a complex disease driven by abnormal genetic and epigenetic alterations, as well as environmental factors. Low levels of genomic methylation in cancer cells were first discovered by Feinberg et al. in 1983 (2). Since then, an increasing number of studies have shown that DNA methylation levels and patterns are disordered with the occurrence and development of tumors. Aberrant DNA methylation, characterized by genome-wide hypomethylation and regional hypermethylation, is common in various cancer forms and is closely associated with tumor initiation and progression (3).

DNA methylation (generating 5-methylcytosine [5mC]) and hydroxymethylation (generating 5-hydroxymethylcytosine [5hmC]) are epigenetic modifications that are frequently aberrant in cancer (4, 5). The conversion of 5mC to 5hmC occurs through an oxidative reaction catalyzed by the ten-eleven translocation (TET) protein family of dioxygenases (6–8). Previous studies have found that both the TET protein family and 5hmC play important roles in tumor development and progression (9). TET2 DNA dioxygenase plays an important role in regulating cell identity and inhibiting tumor development by regulating DNA methylation and the expression of a large number of genes. The expression level of TET2 is decreased in liver cancer, breast cancer, lung cancer, prostate cancer and other solid tumor tissues compared to normal tissues, and this downregulation decreases the content of its catalytic product 5-hmC, which is closely related to tumor development (10, 11). Changes in TET expression at the gene and protein levels and changes in the 5hmC level are thought to be associated with the development and progression of several cancer types, but there are little data related to EC. In this study, we detected the expressions of TET2 and 5hmC and analyzed their clinical significance in endometrial adenocarcinoma to explore the possible mechanism of TET2 in the development of endometrial cancer.

Metabolic diseases, such as central obesity, type 2 diabetes, and polycystic ovary syndrome (PCOS), are risk factors for type I EC. Diabetes increases the risk of EC by 2.8-fold. Metformin, which is safe and economical, is the first choice for treatment of type 2 diabetes. A large number of epidemiological and clinical observations have shown that metformin can reduce the incidence of a variety of tumors, improve the prognosis of patients with coexisting type 2 diabetes and tumors and improve the patient survival rate (12). Therefore, metformin is expected to become a new tumor treatment or adjuvant antitumor drug. Our previous studies have showed that metformin inhibits the proliferation of EC cells, but the exact mechanism remains unclear. Recent studies have revealed that the TET2 phosphorylation pathway mediated by the energy receptor adenosine monophosphate-activated protein kinase (AMPK) plays an important role in linking diabetes and cancer (13). Metformin is known to be an AMPK activator, so could metformin inhibit the proliferation of endometrial cancer by regulating TET2? In this study, we attempted to preliminarily explore the above possibilities through cytological experiments.

## Materials and Methods

### Analysis of TET2 in the TCGA Public Database

The endometrial cancer dataset, including mRNA expression and clinical information, was obtained from The cancer genome atlas (TCGA, https://portal.gdc.cancer.gov/) database. The transcriptome data from TCGA was normalized and analyzed using the Limma package. Student t-test and Kruskal-Wallis test were applied to calculate the significance of expression differences between two or more groups, respectively. The univariate cox regression analysis was used to calculate the association between the expression level of TET2 and patient’s overall survival (OS). Hazard ratios (HRs) and 95% confidence intervals (CIs) were computed based on the Cox regression analysis. Survival curves were estimated using the Kaplan-Meier method and were compared using the log-rank test. The significance was defined as a P value of<0.05.

### Tumor Samples

Approval for patient sample analyses was obtained from the Ethics Committee of Xuzhou Central Hospital Affiliated Xuzhou Medical University. The studies were conducted in accordance with the Declaration of Helsinki. All samples were collected from Xuzhou Central Hospital (Xuzhou, Jiangsu, China). In all, 88 EC tissues and 20 normal endometrial tissues were included in the study. Normal endometrial tissues were obtained from women who were undergoing a hysterectomy (for conditions such as uterine fibroids or prolapse).

### Immunohistochemical Staining

Immunohistochemical staining was performed using 4-μm thick paraffin-embedded tissue blocks. Blocking with 3% hydrogen peroxide was performed for 30 min to quench endogenous peroxidases. Tissue sections on slides were incubated with primary antibody (1:200 dilution) at 4°C overnight. Then, secondary antibody was added and incubated with the tissues at 37°C for 30 min. Diaminobenzidine tetrahydrochloride was used as a chromogen. As a negative control, phosphate-buffered saline (PBS) was used instead of primary antibody. TET2 positivity was visible as yellow-brown granules in the nucleus and cytoplasm, and 5hmC-positive staining was visible as a brownish yellow color in the nucleus. Each section was independently assessed by two pathologists without prior knowledge of patient data. The samples were assigned a mean score considering both the intensity of staining and the proportion of cells with an unequivocal positive reaction in the immunohistochemical analysis. Positive reactions were defined as those showing brown signals mainly in the cell nucleus. The staining index (range, 0-3) was determined according to the staining intensity and positive area. Scores of 0-3 were defined as follows: 0, negative; 1, weak; 2, moderate; and 3, strong. For statistical analysis, scores of 0-1 were considered to indicate low expression, and scores of 2-3 were considered to indicate high expression.

### Cell Culture and Reagents

The EC cell lines Ishikawa and HEC-1-A were purchased from The Cell Bank of Type Culture Collection of Chinese Academy of Sciences (Shanghai, China). The cell lines were cultured in RPMI-1640 (Thermo Fisher Scientific, Inc., Waltham, MA, USA) and McCoy’s 5A (Sigma-Aldrich; Merck KGaA, Darmstadt, Germany) medium containing 10% fetal bovine serum (FBS; Thermo Fisher Scientific, Inc.) at 37°C in an atmosphere of 5% CO_2_. The cells were passaged every 3-5 days. Metformin was purchased from Sigma-Aldrich; Merck KGaA. Primers were purchased from Sangon Biotech Co., Ltd. (Shanghai, China). The anti-phosphorylated (p)-AMPK (cat. no. BS4457P), and anti-AMPK (cat. no. BS4457) antibodies were purchased from Bioworld Technology, Inc. (St. Louis Park, MN, USA). TET2 primary antibodies were purchased from Abcam (USA) (ab94580, ab214728).

### siRNA Transfection

The endometrial carcinoma cell lines were transfected with siTET2 or siControl *via* reverse transfection using Lipofectamine RNAiMAX (Invitrogen, USA). In parallel, 1.5 μL of Lipofectamine RNAiMAX was mixed with 50 μL of Opti-MEM. The solution mixture was mixed by gentle pipetting and incubated for 10-20 min at room temperature to allow siRNA/lipid complexes to form. EC cells were suspended in complete growth medium without antibiotics at 50,000 cells/mL, gently mixed with 100 μL of the transfection solution, and plated. The cells were incubated for 24-72 h at 37°C and then assayed for gene knockdown.

### Cell Proliferation Assay (Cell Counting Kit-8; CCK-8)

The experiment was conducted according to the protocol of the Cell Counting Kit-8 Reagent Kit (Dojindo Molecular Technologies, Inc., Kumamoto, Japan). Cells transfected with siRNAs were seeded at 5,000 cells per well in 96-well plates and the TET2 knockdown cells and control cells were treated with 5mM metformin. They were both incubated in medium containing 10% FBS for 24 h, 48 h, 72 h and 96 h. After changing the medium without metformin, CCK-8 was added to each well, and the plates were incubated at 37°C for 1 h. Absorbance was measured at 450 nm using an automated microplate reader (Infinite 200; Tecan, Männedorf, Switzerland).

### Western Blot Analysis

EC cells (1×10^5^/dish) were plated in 10-cm dishes and treated with 1, 5, or 15 mM metformin for 24 h. The cells were collected, resuspended in cell lysis buffer for western blotting, incubated on ice for 30 min, and centrifuged at 12,000× g for 10 min at 4°C. The supernatant was collected, and the protein content was quantified using a bicinchoninic acid (BCA) protein assay kit (Beyotime). Protein samples (20 µg) were separated in a 10% sodium dodecyl sulfate (SDS)-polyacrylamide gel and transferred to a polyvinylidene difluoride membrane (Millipore). After washing with PBST three times, the membranes were blocked with 5% nonfat milk for 30 min and then incubated with primary antibodies overnight at 4°C. The blots were then washed with PBST three times and incubated with the appropriate secondary antibodies. After washing with PBST three times, the protein bands were detected using an Odyssey Infrared Imaging system (Li-COR Biosciences). Primary antibodies against TET2 and AMPK were used at a dilution of 1:1,000, and secondary antibodies were used at a dilution of 1:2,000. The relative band intensity was analyzed with ImageJ software (version 1.47) (Schneider et al., 2012) and calculated as a ratio relative to the glyceraldehyde 3-phosphate dehydrogenase (GAPDH) band intensity. For evaluation of different blots, each band of the replicates was normalized to the GAPDH band intensity and then averaged. The averaged intensities were used for comparisons.

### Reverse Transcription-Quantitative Polymerase Chain Reaction (RT−qPCR)

Ishikawa and HEC-1-A cells were plated at a concentration of 10^5^ cells/well in 6-well plates for 24 h at 37°C and subsequently treated with metformin (0, 1, 5 and 15 mM). Total RNA was extracted from the harvested EC cells according to the manufacturer’s instructions using TRI reagent (Sigma). The RNA concentration was determined by measuring the OD at 260 nm. First-strand complementary DNA (cDNA) was synthesized with a SuperScript II First-Strand Synthesis System for quantitative reverse transcription-polymerase chain reaction (qRT-PCR; Invitrogen). qPCR amplification was carried out using actin as an endogenous control. SYBR Green probes for each gene were used. The primers are listed in **Table S1**. Real-time PCR was carried out with 50 ng of cDNA and SYBR PCR master mix (TaKaRa) in an Agilent Mx3000P Real-time PCR System using the two-step procedure (95°C 2 min, 1 cycle; 95°C 15 s, 60°C 1 min, 30 cycles). Relative quantitation of the expression of each single gene was performed using the comparative threshold cycle method.

### Quantification of Global DNA Hydroxymethylation (Indicated by 5hmC) *via* ELISA

The extracted genomic DNA was stored at −80°C. Global DNA hydroxymethylation (indicated by 5hmC) was assessed using a MethylFlash Global DNA Hydroxymethylation ELISA Easy kit (colorimetric) from EpiGentek according to the instructions provided by the manufacturer.

### Statistical Analysis

All assays were repeated independently a minimum of three times (n ≥ 3), and three wells per assay were used for each treatment in each cell line. The experimental data are expressed as the mean ± standard deviation (SD). One-way analysis of variance was used for statistical analyses and was performed using SPSS software (version 22.0). Data were compared between the two groups using a least significant difference test. The log-rank test was used to compare differences in the overall survival rate. The Cox proportional hazard regression model was used for multivariate analysis. Statistical significance is indicated by * for *P* < 0.05 and ** for *P* < 0.01.

## Results

### Clinical Significance of Expression of TET2 in Endometrial Cancer From TCGA Public Database

**Figure 1:**
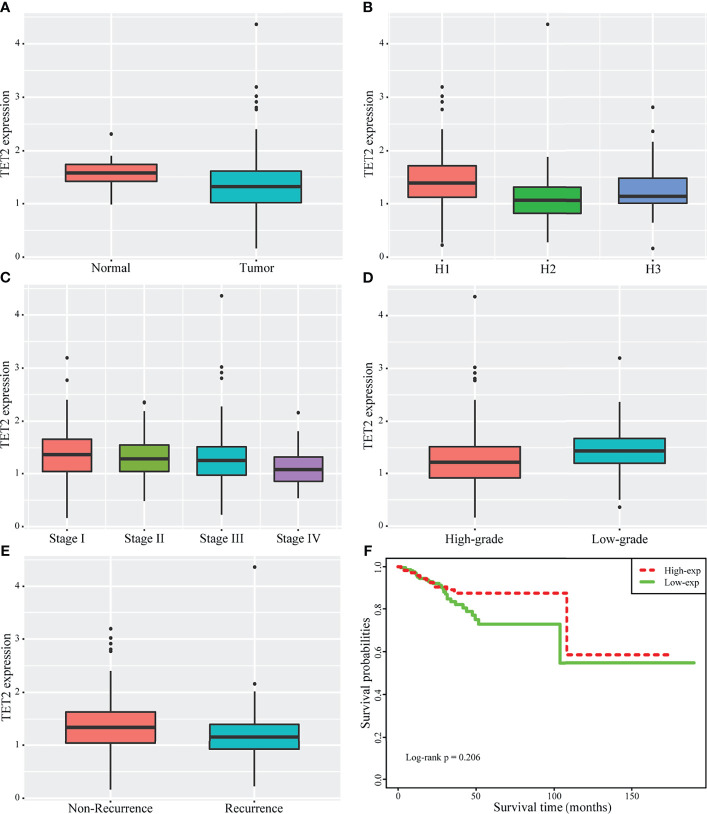
Clinical significance of expression of TET2 in endometrial cancer from TCGA public database. The significant differences of TET2 expression between normal and endometrial cancer **(A)**. TET2 expression was also significantly different among different histopathological samples (1 stands for Endometrioid endometrial adenocarcinoma, 2 stands for Serous endometrial adenocarcinoma, 3 stands for Mixed serous and endometrioid) **(B)**. With the increase of stage, TET2 expression was continuously down-regulated (C: 1 stands for Stage I, 2 stands for Stage II, 3 stands for Stage III, 4 stands for Stage IV). TET2 was significantly down-regulated in the high-grade group **(D)**. TET2 was significantly lower in patients with recurrence **(E)**. TET2 expression is a protective factor, and the high expression group tends to have a better prognosis **(F)**.

The differential expression of TET2 between normal endometrial tissue and endometrial cancer tissue was observed using the endometrial cancer data in TCGA database. TET2 expression was significantly down-regulated in cancer samples (P = 3.250346E-05)(**Figure 1A**). We also compared TET2 expression between different histopathological samples (Endometrioid endometrial adenocarcinoma, Serous endometrial adenocarcinoma, Mixed serous and endometrioid). The results showed that the expression of TET2 was also significantly different among different histopathological samples, and it was significantly over-expressed in Endometrioid endometrial adenocarcinoma (P< 0.05)(**Figure 1B**). With the increase of stage, the expression of TET2 was continuously down-regulated, with significant differences in different stages(P=0.00495) (**Figure 1C**). Besides, TET2 was significantly down-regulated in the high-grade group(P=2.197738E-05) (**Figure 1D**). Comparison of TET2 expression between patients with and without recurrence showed that TET2 expression was significantly lower in patients with recurrence (P=0.0425) (**Figure 1E**). The correlation between TET2 expression and survival was observed by univariate Cox. The results showed that TET2 expression was a protective factor, but there was no significant correlation with overall survival (P =0.378; HR = 0.7257). Using the median expression value of TET2 as the dividing line, patients were divided into the high and low expression group (lower than the median is the green line, and higher than the median is the red line). There was no significant difference in survival between the two groups, but the high-expression group tended to have a better prognosis. (log-rank p = 0.206) (**Figure 1F**).

### TET2 and 5hmC Are Expressed at Low Levels in Endometrial Carcinoma Tissue

**Figure 2:**
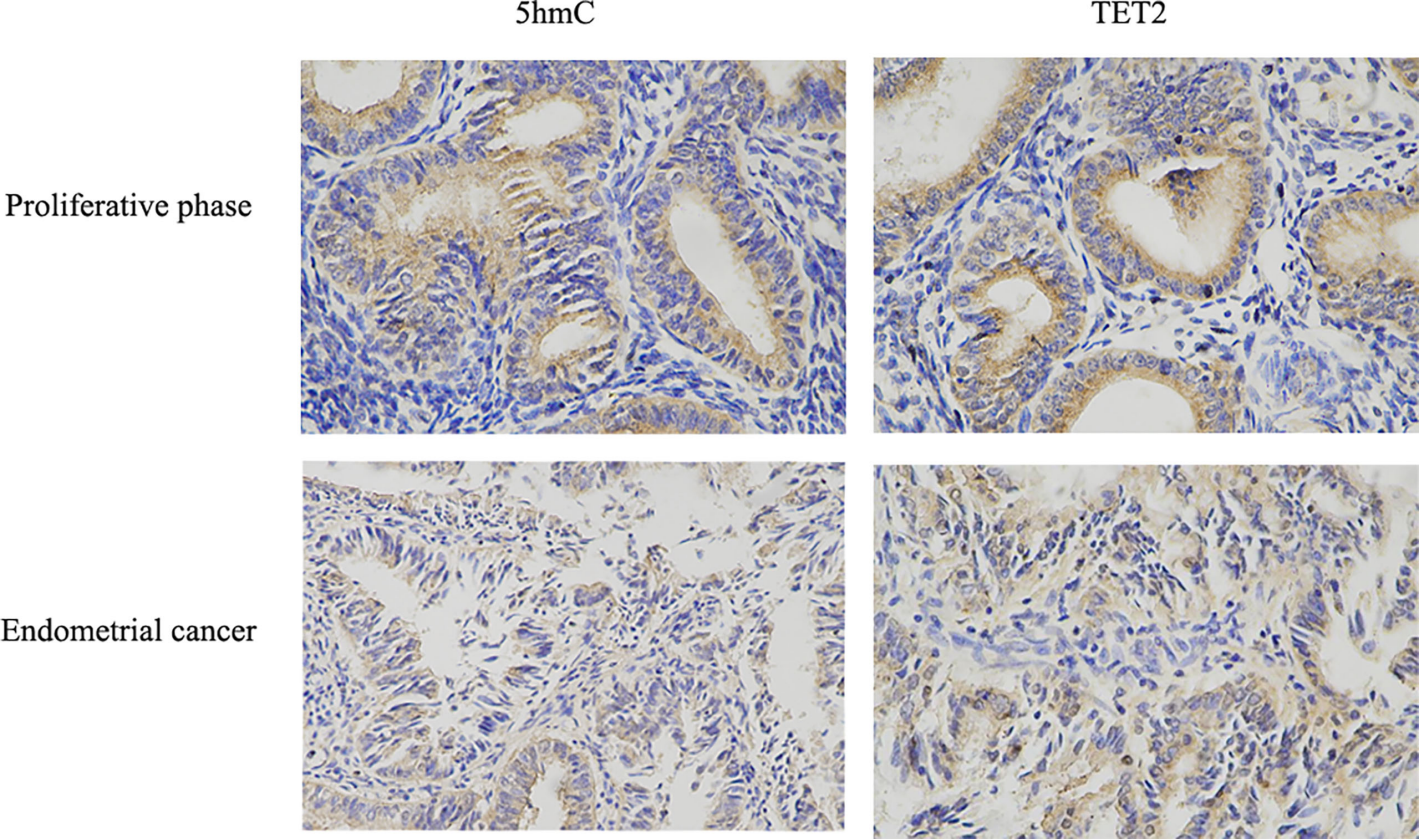
TET2 and 5hmC were low-expressed in endometrial cancer. The expression of TET2 and 5hmC in proliferative phase endometrial tissue and EC tissue was assessed using IHC staining. Representative images were captured at ×400 magnification. TET2 and 5hmC IHC scores in EC tissues compared with proliferative phase endometrial tissues were presented.

TET2 expression among different histopathological samples (Endometrioid endometrial adenocarcinoma, Serous endometrial adenocarcinoma, Mixed serous and endometrioid) from TCGA public database showed that the expression of TET2 was also significantly different among different histopathological samples, and it was significantly over-expressed in Endometrioid endometrial adenocarcinoma (**Figure 1B**). Endometrioid adenocarcinoma accounts for 80-90% of the pathological types of endometrial cancer. Thus, we examined the levels of TET2 and 5hmC in endometrioid endometrial adenocarcinoma tissues. The conversion of 5mC to 5hmC occurs through an oxidative reaction catalyzed by the TET protein family of dioxygenases. We analyzed TET2 expression in a series of 88 endometrial carcinoma samples and 20 normal proliferative endometrium samples *via* immunohistochemistry. TET2 expression was observed in the nucleus and cytoplasm of cells as yellow-brown granules. 5hmC-positive staining was indicated by a brownish yellow color in the nucleus. We found that TET2- and 5hmC-positive staining was present at a higher level in patients with a proliferative endometrium than in patients with EC (**Figure 2**). These data suggest that TET2 may be associated with EC.

### Relationships Between TET2 and 5hmC Expression and Clinicopathological Factors in EC

Correlations between TET2 and 5hmC expression and the clinicopathologic characteristics of endometrial carcinoma are shown in **Table 1**. TET2 expression in endometrial adenocarcinoma was correlated with the degree of differentiation (P < 0.05). The positive TET2 expression rate in poorly differentiated tissues was lower than that in highly differentiated tissues (P < 0.05). The 5hmC level in endometrial adenocarcinoma was associated with clinical stage, differentiation, depth of myometrial invasion, and lymph node metastasis (P < 0.05). The positive rate of 5hmC staining decreased with tumor malignancy (**Table 1**).

**Table 1:** Relationships between TET2, 5-hmC and clinicopathological factors in EC.

Grouping	TET2 positive [n (%)]	χ2	*P*	5-hmC positive [n (%)]	χ2	*P*
FIGO Stage		5.699	0.058		7.234	0.027*
I	40 (60.6)			45 (68.2)		
II	4 (57.1)			2 (28.6)		
III+IV	4 (26.7)			6 (40.0)		
Differentiation		6.145	0.046*		23.839	0.000**
Low	7 (31.8)			5 (22.7)		
Medium	9 (64.3)			6 (42.9)		
High	32 (61.5)			42 (80.8)		
Myometrial invasion		3.024	0.082		11.968	0.001**
<1/2	36 (61.0)			43 (72.9)		
≥1/2	12 (41.4)			10 (34.5)		
Lymph node metastasis		3.364	0.067		4.779	0.029*
No	47 (58.0)			52 (64.2)		
Yes	1 (14.3)			1 (14.3)		

^*^P < 0.05, ^**^P < 0.01.

### Relationships Between TET2 and 5hmC Levels in EC and the Survival Time of Patients

**Figure 3:**
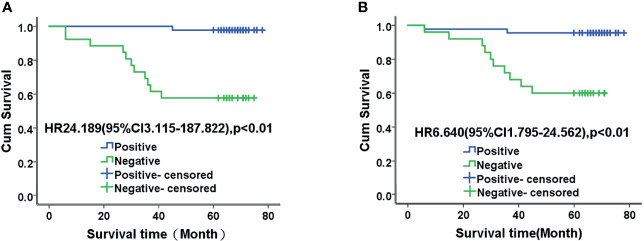
Kaplan-Meier analysis of TET2 **(A)** and 5-hmC **(B)** expression and prognosis in patients with endometrial adenocarcinoma. The mean survival time of patients with negative TET2 and 5hmC staining was significantly shorter than that of patients with positive staining (P < 0.01).

Among the 88 patients with endometrial adenocarcinoma, 58 (65.9%) survived, 12 (13.6%) died, and 18 (20.5%) were lost to follow-up. Surviving patients were followed up for 60 to 89 months, and the patients who died had survival times ranging from 6 to 45 months. The mean survival time of patients was 58.00 ± 18.11 months, and the median was 66 months. The mean and median survival times of patients with positive TET2 expression were 66.57 and 68.22 months, respectively, and those of patients with negative TET2 expression were 46.74 and 44 months, respectively. The mean and median survival times of patients with positive staining for 5hmC were 64.47 and 68.33 months, respectively, and those for patients with negative staining for 5hmC were 48.09 and 46.50 months, respectively. The mean survival time of patients with positive TET2 and 5hmC staining was 63.46 months, and the median survival time was 67.50 months. The mean survival time of patients with negative staining TET2 and 5hmC was 41.35 months, and the median survival time was 36.50 months. The mean survival time of patients with negative TET2 and 5hmC staining was significantly shorter than that of patients with positive staining (P < 0.01). The 5-year survival rates of TET2-positive and TET2-negative patients were 97.7% and 57.7%, respectively, and those of 5hmC-positive and TET2-negative patients were 93.5% and 62.5%, respectively (**Figure 3**).

### Multivariate Cox Regression Analysis of Prognostic Factors in Patients With EC

Multivariate Cox regression analysis revealed that stage, lymph node metastasis, and TET2 expression were associated with prognosis in patients with endometrial adenocarcinoma (P < 0.05). After adjusting for possible confounders, stage, lymph node metastasis, and TET2 expression may be independent prognostic factors in patients with endometrial adenocarcinoma [hazard ratio (HR) = 13.553, 95% confidence interval (CI): 1.509–121.677, P = 0.020; HR = 15.359, 95% CI: 1.284–183.783, P = 0.031; HR = 14.520, 95% CI: 1.060–198.843, P = 0.045] (**Table 2**).

**Table 2:** Multivariate Cox regression analysis of prognostic factors in patients with EC.

Index	B	SE	Wald	*P*	HR	95% CI
Stage (ref = I)			6.024	0.049*		
II	3.214	1.87	2.954	0.086	24.875	0.637–971.197
III+IV	2.607	1.12	5.418	0.02	13.553	1.509–121.677
Differentiation (ref = low)			2.788	0.248		
Medium	-0.525	1.114	0.222	0.637	0.591	0.067–5.249
High	-2.209	1.335	2.737	0.098	0.11	0.008–1.504
Myometrial invasion	-2.92	1.557	3.519	0.061	0.054	0.003–1.140
Lymph node metastasis	2.732	1.266	4.653	0.031*	15.359	1.284–183.783
TET2	2.676	1.335	4.015	0.045*	14.52	1.060–198.843
5-hmc	9.773	115.514	0.007	0.933	17551.949	0.000–3.712

^*^P < 0.05.

### Anticancer Effects of TET2 in EC Cell Lines

**Figure 4:**
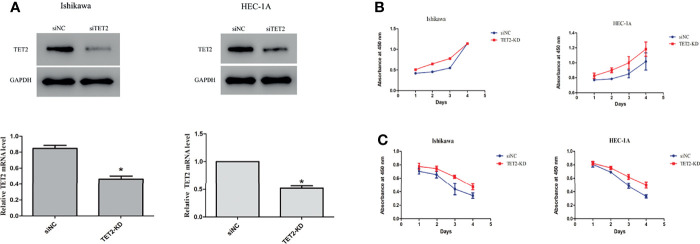
Anticancer effects of TET2 in EC cell lines. The TET2 knockdown efficiency of the gene-specific siRNA was confirmed using western blotting and real-time PCR **(A)**. Knockdown of TET2 increased the proliferationin of EC cell lines **(B)**. The TET2 knockdown cells and control cells were treated with 5 mM metformin. The knockdown of TET2 attenuated the inhibitory effect of metformin on the proliferation of EC cells **(C)**. ^*^P < 0.05, compared with control.

To explore the role of TET2 in EC cell lines, we knocked down TET2 using siRNA. The TET2 knockdown efficiency of the gene-specific siRNA was confirmed using real-time PCR and western blotting (**Figure 4A**). Knockdown of TET2 gene expression with siRNA significantly increased the proliferation rate of Ishikawa (P<0.01) and HEC-1-A (P<0.01) cells compared with that of cells transfected with nontargeting siRNA (**Figure 4B**). In addition, we further examined the effect of TET2 on metformin-mediated inhibition of EC cell proliferation. TET2 knockdown cells were treated with 5 mM metformin for 72h, and the results showed that knockdown of TET2 attenuated the inhibitory effect of metformin on EC cell proliferation (**Figure 4C**).

### Metformin Increased TET2 and 5hmC Expression in EC Cells

**Figure 5:**
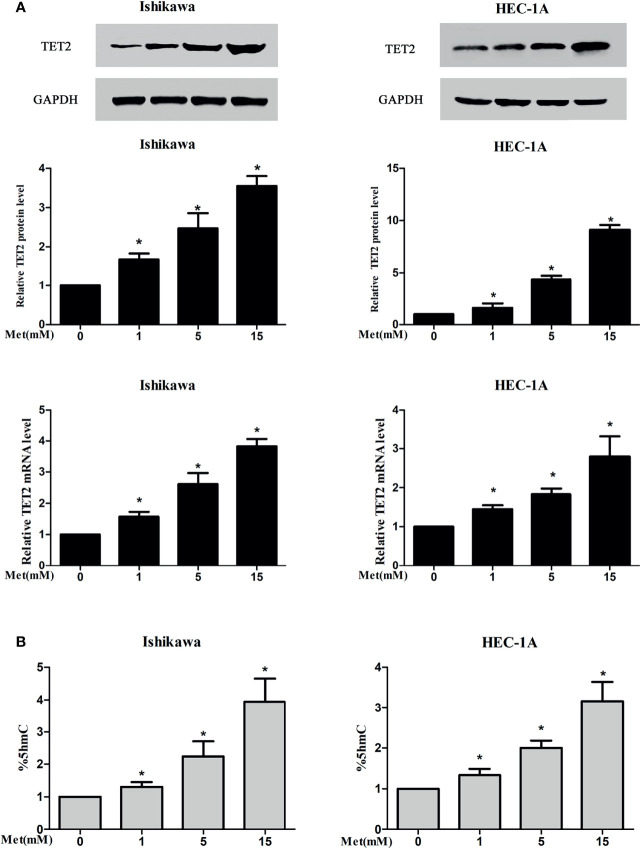
Metformin increased the expression of TET2 and 5hmC in EC cells. Two types of EC cells were treated with metformin at different concentrations for 24 h The expression of TET2 was detected by western blotting and real-time PCR and 5hmC was detected by ELISA. Metformin increased the protein and mRNA expression of TET2 in a dose-dependent manner **(A)**. Metformin also increased the level of 5hmC in a dose-dependent manner **(B)**. ^*^P < 0.05, compared with control.

To examine the potential regulation of the expression and activation of TET2 and its substrates by metformin in endometrial carcinoma, two types of EC cells were treated with metformin at different concentrations for 24 h. In our study, western blotting and real-time PCR results showed that metformin treatment resulted in a potent increase in TET2 protein and mRNA expression, which occurred in a dose-dependent manner (**Figure 5A**). Metformin also increased the 5hmC level in a dose-dependent manner (**Figure 5B**).

### TET2 and 5hmC Regulation by Metformin Depends on the Presence of AMPK

**Figure 6:**
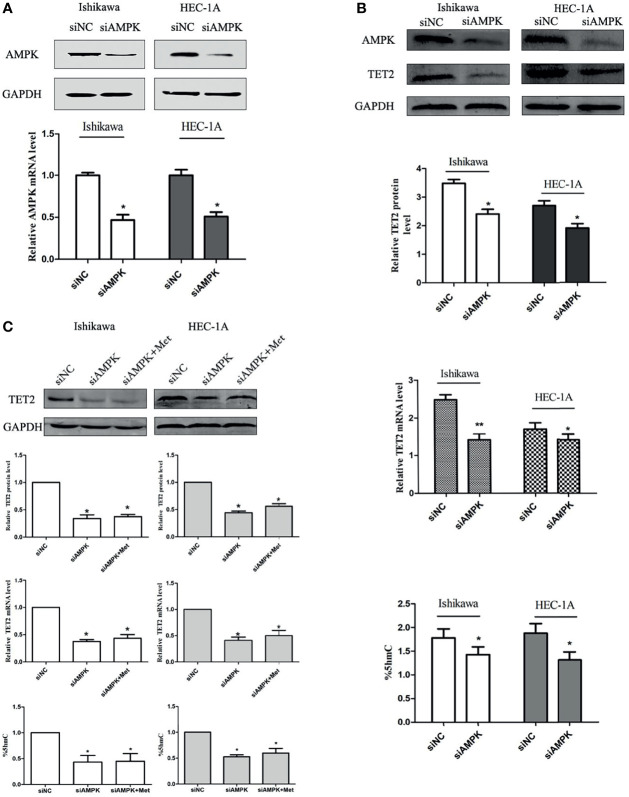
The regulation of TET2 and 5hmC by metformin depends on the presence of AMPK. We knocked down AMPK using an siRNA. The AMPK knockdown efficiency of the gene-specific siRNA was confirmed using western blotting and real-time PCR **(A)**. The knockdown of AMPK gene expression with the siRNA significantly reduced the expression of TET2 and the level of 5hmC **(B)**. Western blotting and real-time PCR results showed that metformin did not significantly increase the expression of TET2 and 5hmC in these cells when knocked down AMPK **(C)**. ^*^P < 0.05, **P < 0.01, compared with control.

Metformin is known as a traditional AMPK activator. Therefore, we further tested whether AMPK is involved in the regulation of TET2 by metformin. We knocked down AMPK using siRNA. The AMPK knockdown efficiency of the gene-specific siRNA was confirmed using real-time PCR and western blotting (**Figure 6A**). At 24 h after siRNA knockdown of AMPK gene expression, TET2 expression and 5hmC level were significantly reduced (**Figure 6B**). Western blotting and real-time PCR results showed that metformin did not significantly increase TET2 or 5hmC expression in these cells after 72 hours of metformin treatment (**Figure 6C**). Thus, we speculated that metformin could regulate TET2 expression through the AMPK pathway.

## Discussion

According to a number of epidemiological studies, endometrial cancer (EC) is associated with chronic exposure to high levels of estrogen (14). However, beyond the involvement of estrogen, the mechanism of carcinogenesis in the endometrium remains unclear. In recent years, there has been a focus on epigenetic mechanisms, which involve regulation of gene expression through chromatin modification without a change in the DNA sequence. Aberrant DNA methylation plays an important role in tumor development, and disorder of DNA demethylation mediated by the ten-eleven translocation (TET) family is an important factor leading to DNA methylation imbalance. DNA 5-hydroxymethylcytosine (5hmC) is a major oxidation product of DNA 5-methylcytosine (5mC), and this reaction is catalyzed by the TET family of dioxygenases (6). To the best of our knowledge, little studies have evaluated the role of TET2 in EC development and effect of metformin on TET2 expression. The results of the present study suggested that TET2 was associated with EC development and could inhibit EC cell proliferation. In addition, we found that metformin could increase TET2 protein expression through AMPK pathway in EC cells.

TET proteins, including TET1, TET2 and TET3, are α-ketoglutarate and Fe2+-dependent enzymes that can oxidize 5mC to 5hmC, which is an epigenetic DNA modification process (6, 15). DNA methylation (generating 5mC) and hydroxymethylation (generating 5hmC) are common epigenetic modifications in cancer (4, 5, 16). Previous studies have found that both the TET protein family and 5hmC play important roles in tumor development and progression (17). TET2 was first identified as a tumor suppressor gene in myelodysplastic syndrome (18). TET2 expression is decreased in liver cancer, breast cancer, lung cancer, prostate cancer and other solid tumors, leading to a decrease in the content of its catalytic product 5hmC, which is closely related to tumor development (10, 11, 19). The results of the present study suggest that the TET2 and 5hmC levels in EC tissues are significantly decreased compared with those in normal endometrial tissues from TCGA public database and immunohistochemical analysis. Our study showed a positive correlation between the 5hmC level and TET2 expression. Similar to the results of most studies of other malignant tumors, the results of our EC studies showed decreased TET2 and 5hmC expression in cancer tissues.

In melanoma, a low 5hmC level is a marker of a poor prognosis and is associated with dysplastic cytomorphological features and tumor progression (20). In solid tumors, low 5hmC levels indicate poor overall survival and a high cumulative recurrence rate (21). In addition, 5hmC levels are highly correlated with tumor stage (22). In terms of the molecular mechanism, 5-hmC is a product of DNA demethylation of TET2, suggesting that loss of TET2 leads to loss of 5-hmC, which can promote cancer occurrence and progression by affecting gene expression patterns. The results of the present study showed that the TET2 expression rate in poorly differentiated EC tissues was lower than that in well-differentiated EC tissues. TET2 expression was significantly reduced in high-grade, advanced, and recurrent endometrial carcinoma from TCGA database. The expression of 5hmC in endometrial adenocarcinoma is related to clinical stage, the degree of differentiation, the depth of muscular infiltration and lymph node metastasis. The level of TET2 and 5hmC is widely decreased in cancer cells and can be used as a marker of the degree of cancer malignancy.

Alterations in genomic 5hmC levels and TET dioxygenase expression are closely associated with the survival rate of cancer patients (11, 23, 24) and are involved in breast (25), prostate, liver (26), lung, pancreatic, colorectal, gastric, small intestine, brain, kidney, and skin cancer and myeloid diseases (27–29). Our results also showed that the 5-year survival of EC patients with negative TET2 and 5-hmC staining was significantly reduced. Multivariate COX regression analysis revealed that TET2 might serve as an independent prognostic factor in patients with endometrial adenocarcinoma, and may be useful in predicting therapeutic effects. These above results suggest that TET2 may play an important role in EC development.

To further investigate the role of TET2 in EC, we assessed the proliferation of Ishikawa and HEC-1-A cells after TET2 knockdown. The results indicated that TET2 knockdown increased EC cell growth, suggesting that TET2 can inhibit EC cell proliferation. DNA methylation plays a key role in the regulation of genes involved in cell growth, proliferation and apoptosis in endometrial tissue (30). Thus, deregulation of the DNA methylation pattern can disrupt cell homeostasis in the endometrium and result in EC development (31). A recent study of colorectal cancer (CRC) indicated that genes with 5hmC in their promoters resist DNA hypermethylation, highlighting the important role that 5hmC plays in cancer cell proliferation (32). However, the mechanism by which TET2 deletion increases the proliferation of EC cells remains to be further studied.

Diabetes is a known risk factor for EC. TET2 is an important link between diabetes and cancer. Glucose-regulated phosphorylation of TET2 by AMPK reveals a pathway linking diabetes to cancer (13). Metformin is a first-line drug for diabetes treatment and has an antiproliferative effect on many types of cancer cells. Our previous studies have shown that metformin inhibits the proliferation of EC cells (33). We speculated that TET2 is involved in the inhibitory effect of metformin on EC cell proliferation. Then, we compared the proliferation of TET2 knockdown cells with that of control cells after metformin treatment. We found that TET2 knockdown significantly inhibited the antiproliferative effect of metformin on EC cells. Therefore, we hypothesized that metformin may inhibit EC cell proliferation by regulating TET2 expression. To test this hypothesis, we examined TET2 expression in EC cells treated with metformin at different concentrations. Our results suggested that metformin can increase TET2 expression and the 5hmC level in a dose-dependent manner. Adenosine monophosphate-activated protein kinase (AMPK) is a highly conserved protein in mammalian cells and a “metabolism and energy receptor” of cells (34). Metformin is a traditional AMPK activator. Metformin inhibits the growth of ECC-1 cells and Ishikawa cells in a dose-dependent and time-dependent manner by activating AMPK and inhibiting the mTOR signaling pathway (35). Metformin failed to inhibit the proliferation of EC cells treated with AMPK siRNA or inhibitors (36). Two potential AMPK catalytic sites were identified by amino acid sequence analysis of TET2, and the TET2 protein was confirmed to be a substrate of AMPK. Activated AMPK can phosphorylate TET2 at serine 99, thus maintaining the stability of the TET2 protein (13). Our results showed that knockdown of AMPK gene expression with siRNA significantly reduced TET2 expression and the 5hmC level and attenuated the inhibitory effects of metformin on these factors. Therefore, we speculate that metformin may regulate TET2 expression by activating AMPK.

## Conclusions

In summary, our current findings demonstrate the expression pattern and clinical significance of TET2 in EC. TET2 can repress EC cells proliferation. In addition, TET2 could be a useful biomarker for predicting the prognosis of EC patients and may represent a novel therapeutic target for EC treatment. Metformin increased TET2 expression and the 5hmC level. The results of the present study reveal that metformin regulates TET2 protein expression by activating AMPK. Our study provides new insight into the antiproliferative effects of metformin in EC.

## Data Availability Statement

The original contributions presented in the study are included in the article/**Supplementary Material**. Further inquiries can be directed to the corresponding authors.

## Ethics Statement

The studies involving human participants were reviewed and approved by Biomedical Research Ethics Review Committee of Xuzhou Central Hospital. The patients/participants provided their written informed consent to participate in this study.

## Author Contributions

JZ and BZ contributed to the conception and design of the study. LK provided access to tissue and prepared tissue samples. YYL, QW, and HX provided clinical information. XZ, JL, and JZ performed cell culture, PCR and western blot. LK and QW carried out cell proliferation experiments. JZ and YL analyzed the data. YL performed TCGA data analysis. JZ and YL prepared the figures and drafted the manuscript. All authors read and commented on the manuscript and approved the final version.

## Funding

This work was supported by the Natural Science Foundation of Jiangsu Province [No. BK20171173]; the Jiangsu Science and Technology planning project [No. BE2019636]; the Science and Technology Project of Xuzhou Health Commission [No. XWKYHT20200039]; and Jiangsu Key Laboratory of New Drug Research and Clinical Pharmacy [No. XZSYSKF2020023].

## Conflict of Interest

The authors declare that the research was conducted in the absence of any commercial or financial relationships that could be construed as a potential conflict of interest.

## Publisher’s Note

All claims expressed in this article are solely those of the authors and do not necessarily represent those of their affiliated organizations, or those of the publisher, the editors and the reviewers. Any product that may be evaluated in this article, or claim that may be made by its manufacturer, is not guaranteed or endorsed by the publisher.
