# Supplementary material for: Metformin Regulates TET2 Expression to Inhibit Endometrial Carcinoma Proliferation: A New Mechanism
Source: Front Oncol. 2022 Apr 11;12:856707. doi: 10.3389/fonc.2022.856707 (PMC9035737; doi:10.3389/fonc.2022.856707)
Supplement: Supplementary file 1 [file Table_1.pdf]

Table S1 The sequences of Primers

Genen	Sequence (5' -> 3')
TET2	
Forward Primer	GATAGAACCAACCATGTTGAGGG
Reverse Primer	TGGAGCTTTGTAGCCAGAGGT
AMPK	
Forward Primer	TTGAAACCTGAAAATGTCCTGCT
Reverse Primer	GGTGAGCCACAACTTGTTCTT
GAPDH	
Forward Primer	GGAGCGAGATCCCTCCAAAAT
Reverse Primer	GGCTGTTGTCATACTTCTCATGG
